# BMP2 Genetically Engineered MSCs and EPCs Promote Vascularized Bone Regeneration in Rat Critical-Sized Calvarial Bone Defects

**DOI:** 10.1371/journal.pone.0060473

**Published:** 2013-04-02

**Authors:** Xiaoning He, Rosemary Dziak, Xue Yuan, Keya Mao, Robert Genco, Mark Swihart, Debanjan Sarkar, Chunyi Li, Changdong Wang, Li Lu, Stelios Andreadis, Shuying Yang

**Affiliations:** 1 Department of Oral Biology, University at Buffalo, The State University of New York, Buffalo, New York, United States of America; 2 Developmental Genomics Group, New York State Center of Excellence in Bioinformatics and Life Sciences, University at Buffalo, The State University of New York, Buffalo, New York, United States of America; 3 Department of Stomatology, The 4th Affiliated Hospital of China Medical University, China Medical University, Shenyang, Liaoning, China; 4 Department of Chemical and Biological Engineering, University at Buffalo, The State University of New York, Buffalo, New York, United States of America; 5 Department of Biomedical Engineering, University at Buffalo, The State University of New York, Buffalo, New York, United States of America; 6 Department of Orthopaedic, Chinese people's liberation army general hospital, Beijing, China; 7 Department of Oral and Maxillofacial Surgery, School of stomatology, China Medical University, Shenyang, Liaoning, China; University of Notre Dame, United States of America

## Abstract

Current clinical therapies for critical-sized bone defects (CSBDs) remain far from ideal. Previous studies have demonstrated that engineering bone tissue using mesenchymal stem cells (MSCs) is feasible. However, this approach is not effective for CSBDs due to inadequate vascularization. In our previous study, we have developed an injectable and porous nano calcium sulfate/alginate (nCS/A) scaffold and demonstrated that nCS/A composition is biocompatible and has proper biodegradability for bone regeneration. Here, we hypothesized that the combination of an injectable and porous nCS/A with bone morphogenetic protein 2 (BMP2) gene-modified MSCs and endothelial progenitor cells (EPCs) could significantly enhance vascularized bone regeneration. Our results demonstrated that delivery of MSCs and EPCs with the injectable nCS/A scaffold did not affect cell viability. Moreover, co-culture of BMP2 gene-modified MSCs and EPCs dramatically increased osteoblast differentiation of MSCs and endothelial differentiation of EPCs *in vitro*. We further tested the multifunctional bone reconstruction system consisting of an injectable and porous nCS/A scaffold (mimicking the nano-calcium matrix of bone) and BMP2 genetically-engineered MSCs and EPCs in a rat critical-sized (8 mm) caviarial bone defect model. Our *in vivo* results showed that, compared to the groups of nCS/A, nCS/A+MSCs, nCS/A+MSCs+EPCs and nCS/A+BMP2 gene-modified MSCs, the combination of BMP2 gene -modified MSCs and EPCs in nCS/A dramatically increased the new bone and vascular formation. These results demonstrated that EPCs increase new vascular growth, and that BMP2 gene modification for MSCs and EPCs dramatically promotes bone regeneration. This system could ultimately enable clinicians to better reconstruct the craniofacial bone and avoid donor site morbidity for CSBDs.

## Introduction

Critical-sized bone defects (CSBDs) are the defects with the minimum length that cannot be spontaneously bridged and that should result in the formation of fibrous connective tissue rather than bone when left untreated [1]. Clinical therapies of CSBDs represent a great challenge for orthopedic and craniomaxillofacial surgeons, because current treatments rely on grafting materials such as autografts, allografts, xenografts, and alloplasts, while all those materials have specific limitations. For example, autograft is considered as the gold standard for bone regeneration because of its properties: osteogenetic, osteoinductive and osteoconductive. However, major concerns over the use of autografts include the additional surgical procedure, insufficient supply, and the morbidity associated with the donor site [2]. Allografts are commonly-used grafting materials and are well documented in experimental and clinical studies [3]. Although improved healing has been reported [4], clinical studies show limited new bone formation [5]. Allografts and xenografts also carry the risk of pathogen transmission and immune rejection [6]. In addition, the life-long immune suppression required following their use is inconvenient to the patients and prone to complications. Alloplastic materials (alloplasts) are not biologically functional and carry the risk of long term foreign body reaction and associated complications [7]. Moreover, various other disadvantages such as multiple surgeries, facial scar formation, and non-union, significantly limit the repair of CSBDs.

Over the last decade, stem cells-based bone repair and regeneration has been extensively studied and become one of the most promising modalities in the animal models and clinical studies[8], [9]. Although the treatment of bone defects using MSCs or genetically modified MSCs can effectively promote bone regeneration in human and animal models [10]–[13], the size of the regenerated bone has been limited for CSBDs repair due to a lack of vessels in the grafts, which prevents sufficient nutritional support to the entire bone graft in such studies. Accumulating evidence has shown that in the complex process of bone formation, an important aspect *in vivo* is vascularization [14]. Both intramembranous and endochondral ossification appear to be associated with blood capillaries [15]. At the site of new bone formation, osteoblasts and osteoprogenitors are located adjacent to endothelial cells in blood vessels, suggesting that angiogenesis and osteogenesis are mutually interdependent [16]. Bone tissue has the unique capacity to regenerate sub-critical-sized defects without fibrosis. This healing capacity relies on the presence of vasculature near the injury site and the ability to recruit progenitor cells to the injury site. This process becomes limited in the case of traumatic injuries and CSBDs, in which significant loss of tissue and vasculature leads to acute necrosis and hypoxia of the surrounding tissue [17]. Thus, promoting vascularization in CSBDs is necessary for regenerating bone *in vivo* [18].

One potential approach to achieve such vascularization is the application of endothelial progenitor cells (EPCs), which have been shown to initiate and facilitate neovascularization [19]. These progenitor cells represent a small cell population with the capability to proliferate, migrate, and differentiate into cells that line the lumen of blood vessels [20]. EPCs have been implanted into various ischemic tissue models, e.g. ischemic hindlimbs [21], areas of myocardial infarction and engineered blood vessels [22]. Moreover, EPCs were shown to contribute to neovascularization and new bone formation in fracture healing [23]. Most recently, the combination of MSCs and EPCs has been used in a study of fracture healing in rats, which showed that the combination significantly promotes bone regeneration compared with using MSCs or EPCs alone [24]. However, this combination still cannot completely heal CSBDs. A major reason is the lack of sufficient integration of biomaterials design, growth factor, and progenitor cells such as stem cells to promote vascularized bone regeneration.

Bone morphogenic proteins (BMPs) are potent morphogens that regulate embryonic development and stimulate bone formation in developed tissues [25]–[27]. BMPs also stimulate angiogenesis through the production of VEGF by osteoblasts [28]. Treatment of EPCs with rhBMP-2 did not induce any significant changes in EPC viability but induced a dose-dependent activation of chemotaxis [28]. BMP2 functions and positive effects for bone repair have been demonstrated in clinical trials and human orthopedic applications [29], [30]. However, the administration of BMPs during orthopedic applications is complicated by its short biological half-lives, localized actions and rapid local clearance [31]–[33], whereas the stable release of BMP2 in biomaterials is still a major challenge [34]. Hence, BMP2- genetically engineered stem cells is considered a very promising alternative for the repair of CSBDs.

In this present study, we aimed to improve the processes of vascularization and osteogenesis in the graft and accelerate ectopic bone formation by integrating the injectable and porous nCS/A scaffold with BMP2 genetically modified MSCs and EPCs to develop a novel multi-stem cell-mediated reconstructive graft for healing CSBDs. We first identified the phenotypes of MSCs and EPCs and analyzed the effect of the injectable nCS/A scaffold on cell viability of MSCs and EPCs. Then we defined whether co-culture of BMP2 gene-modified MSCs and EPCs can increase osteoblast differentiation of MSCs and endothelial differentiation of EPCs *in vitro*. We further tested the multifunctional bone reconstruction system consisting of an injectable and porous nCS/A scaffold (mimicking the nano-calcium matrix of bone) and BMP2-genetically engineered MSCs and EPCs in a rat critical-sized (8 mm) caviarial bone defect model. Our results demonstrated that EPCs increase new vascular growth, and that BMP2 gene modification for MSCs and EPCs dramatically promotes vascularized bone regeneration.

## Materials and Methods

### Preparation of injectable nCS/A scaffold

nCS was produced according to the method of Park et al [35], [36]. Briefly, a cryo-vacuum process was used to convert calcium sulfate dihydrate microparticles into calcium sulfate dihydrate nanocrystals, then subjected to oven drying to obtain calcium sulfate hemihydrate nanoparticles (nCS) as described previously [37]. Sterilization was performed by glow discharge treatment (GDT). The injectable nCS/A pastes were formulated using nCS and alginate as described in[38]. Briefly, alginate was dissolved in PBS to prepare a 10% solution (w/v) and then the pH was adjusted to 7.2 –7.4. 135 mg nCS powder was mixed with 150 µl alginate. The total mass of nCS and alginate was 150 mg. After 2 min following the mixing, 25 µl cell suspension with 1×10^6^ MSCs, EPCs or MSCs+EPCs (1:1) infected with Ad-*LacZ* or Ad-*BMP2* (multiplicity of infection or MOI: 100) was respectively added to the nCS/A paste to generate an injectable scaffold. All operations were performed at 4°C.

### Preparation and culture of MSCs and EPCs

Animal procedures were conducted in accordance with the protocol approved by IACUC of the University at Buffalo. Sprague-Dawley (SD) rats, 6–8 week- old, were euthanized by CO_2_. The rat femurs and tibias were dissected from the surrounding tissue in sterile hood. Metaphyses from both ends were resected and bone marrow was collected by flushing the diaphysis with PBS. The MSC expansions were performed as described previously [39]. Briefly, bone marrow derived mononuclear cells (BMNCs) were collected after separating bone marrows by Histopaque-1083 (1.077 g/mL; Sigma) density gradient centrifugation at 400 *g* for 20 min, and then washed twice with Dulbecco's phosphate-buffered saline supplemented with 2 mM EDTA (DPBS-E). BMNCs were cultured in the complete media (CM media, which is DMEM medium (Gibco), supplemented with 10% fetal bovine serum (FBS, Gibco), L-glutamine (2 mmol/L), and penicillin (100 U/mL)). Non-adherent cells were removed with fresh CM media at day 3, and then the media was changed every two days. After 5–7 days, the adherent cells were released using 1×TrypLE™ (Invitrogen) and reseeded onto tissue culture flasks for subsequent passages.

The preparation and culture of EPCs were performed as described [40], [41]. Briefly, BMNCs were washed twice with PBS, and then suspended in EGM media (EGM-2 medium supplemented with growth factor bullet kit (Lonza, Cologne, Germany)) at a density of 1×10^6^ cells/ml. After 24 h incubation at 37°C, 5% CO_2_, nonadherent cells were removed and fresh EGM media was added to the culture dishes. The media was changed every 3 days. After 5–7 days, the adhered cells with 90% confluence were split for subsequent passages.

### BMP2 gene transfer

BMP2 adenovirus was produced as previously described [42]. Viral titers were estimated by optical density and the standard plaque assay as described previously [43]. These preparation methods produced 1.8×10^10^ particles/ml Ad-BMP2 viruses. For transduction of MSCs and EPCs, Ad-LacZ (as a control) or Ad-BMP2 adenovirus at MOI of 100 was added to cells in serum-free medium. After 4 h, serum was added to a final concentration of 2%, and cells were cultured for an additional 24 h. Cells were then transferred to osteogenic (OS) media (complete media supplemented with 50 µg/ml ascorbic acid, 10^−8^ M dexamethasone and 10 mM sodium β-gylcerol-phosphate) [43] and/or EGM media and fed every 2 days unless indicated otherwise.

### Flow Cytometry Analysis and immunofluorescence staining

MSCs and EPCs were harvested at passage 4, 1×10^6^ cells were washed with 10% FBS/PBS and centrifuged at 1000 rpm, 5 min to gather a pellet. For flow cytometry analysis, MSCs were stained with FITC-conjugated rat anti-CD44, Cy5.5-conjugated rat anti-CD90, PE-conjugated rat anti-CD31 and Alexa Fluor 647-conjugated rat anti-CD34 antibodies at a concentration of 2 µg/ml at 4°C. EPCs were stained with PE-conjugated rat anti-CD31, Alexa Fluor 647-conjugated rat anti-CD34, FITC-conjugated rat anti-CD45 and FITC-conjugated rat anti-CD133, at a concentration of 2 µg/ml at 4°C. Mouse IgG was served as negative controls. Unbound antibody was washed with 2 ml of 10% FBS/PBS after 30 min. Then pellets were re-suspended in 500 µl PBS and examined by flow cytometry with 10,000 events recorded for each condition. The results were analyzed by FACS Express software. For immunofluorescence staining [44], EPCs from passage 3 or 4 were co-stained with DPBS-E containing 10 µg/ml DiI-labeled acLDL or FITC-conjugated Lectin (Biomedical Technologies) for 1 h at 37°C, then observed using fluorescence microscopy.

### Co-culture of MSCs and EPCs *in vitro*

To define the best ratio of EPCs and MSCs for bone regeneration, seven groups were divided. These groups were MSCs alone, EPCs alone, and EPCs: MSCs at ratios of 2:1, 1:1, 1:2, 1:5 and 1:10. Cells were plated at the cell number of 1×10^5^ cells per well in 12-well plates and induced with EGM/CM media (EGM media: complete media ratio of 1:1) or EGM/OS media (EGM media: OS media ratio of 1:1) for 7 days for ALP activity assay as previously described[43].

After optimizing the co-culture ratio of MSCs and EPCs and cell media, we further compared the role of BMP2 in MSCs, EPCs and co-cultured MSCs and EPCs. MSCs and EPCs with or without BMP2 gene modification were mixed at 1:1 ratio based on the result from above analysis. Totally, eight groups were tested. These groups were MSCs alone (M), EPCs alone (E), MSCs +EPCs (M+E), BMP2 gene-modified MSCs (B2/M), BMP2 gene-modified EPCs (B2/E), EPC+BMP2 gene-modified MSCs (B2/M+E), MSC+BMP2 gene-modified EPCs (B2/E+M), and BMP2 gene-modified MSCs+BMP2 gene-modified EPCs (B2/(M+E)). BMP2 gene-modified EPCs or MSCs were generated with Ad-BMP2 as descripted in the *BMP2 gene transfer section*. Cells were plated at 5×10^4^ cells per well in 24 - well plates. The cells were induced in EGM/CM media (1:1) for 7 days for ALP activity assay and 14 days for qRT-PCR analysis.

### MTS Cell Viability Assay

The cell viability was assayed using the MTS cell viability assay kit (Promega). There were four groups. 1. Plating: 1×10^4^ cells were directly plated into each well of the 96-well plate. 2. Mixing group (Mix): 13.5 mg nCS was mixed with alginate at the ratio of 90:10 in an ice-cold 96-well plate as described in the Preparation of injectable nCS/A scaffold section. After 2 min, 1×10^4^ cells were mixed with the paste. 3. Mixing+injection group (mix+Inj): the 135 mg nCS was mixed with alginate at the ratio of 90:10 in an ice-cold 35 mm plate. After 2 min, 1×10^5^ cells were mixed with the paste and 1/10 of the total volume of the mixture was manually transferred into a 1 ml syringe. 1/10 of the total volume of the mixture was injected into each well of the 96-well plate. 4. Seeding group (Seeding): 13.5 mg nCS was mixed with alginate at the ratio of 90:10 in an ice-cold 96-well plate as described in the Preparation of injectable nCS/A scaffold section, then put in room temperature for 1 h to form the preformed scaffold for each well. The preformed scaffolds were pre-wetted in CM media for 2 h. Then 1×10^4^ cells for each well were seeded on the preformed scaffold. In each group, 1×10^4^ cells were placed in each well, and 5 wells were used. After induced with OS media for the indicated time, the cells were incubated with 100 µl OS media and 20 µl MTS assay reagent for an additional 3 h. Finally, the supernatants were transferred to a new 96 well plate for recording the absorbance at 490 nm using a 96-well plate reader.

### Alkaline phosphatase activity assay

ALP activity was determined by using an ALP assay kit (Sigma) following the manufacturer's instructions. Briefly, cells were added to an alkaline buffer solution (1.5 M, pH10.3) containing 10 mM p-nitrophenyl phosphate as a substrate. NaOH solution (3 M) was used as stop solution, and optical density was determined at 405 nm [43]. ALP activity was normalized by the DNA content and expressed as nmol of p-nitrophenol produced per minute per mg of total DNA. [45]. For the implanted samples, the samples were harvested and smashed in liquid nitrogen, and lysed in 1 ml harvest buffer for 1 hour, and then the samples were homogenized at low power with homogenizer to further lyse cells. After being centrifuged at 2000 rpm for 10 min, 10 µl supernatant were taken for ALP activity assay.

### Quantitative reverse transcription-polymerase chain reaction (qRT-PCR)

Total RNAs were isolated using Trizol reagent (Invitrogen) according to the manufacturer's instructions. Reverse transcription of total RNA was carried out using the SuperScript^™^ first strand synthesis system for RT-PCR [46] (Invitrogen). Synthesized cDNA was used to perform real time-PCR reactions[45]. PCR amplifications were performed using the specific primers for analyzing the expression of osteoblast marker genes including *osteocalcin (OCN), collagen Type I, Alpha 1 (Col1α1), BMP2* and endothelial marker genes including *vascular endothelial growth factor* (*VEGF), Cadherin (cdh5)* and *von Willebrand factor* (*vWF).* The primers for these genes are*: OCN* (forward primer, 5′- TCTTTCTCCTTTGCCTGGC -3′; reverse primer, 5′- CACCGTCCTCAAATTCTCCC -3′); *Col1α1* (forward primer, 5′- GCA ACA GTC GCT TCA CCT ACA -3′; reverse primer, 5′- CAA TGT CCA AGG GAG CCA CAT -3′); *BMP2* (forward primer, 5′- TCCGCTCCACAAACGAGAAA -3′; reverse primer, 5′- AAAGGCATGATAGCCCGGAG -3′); *VEGF* (forward primer, 5′- CCGAAACCA TGAACTTTCTGC -3′; reverse primer, 5′- GACTTCTGCTCTCCTTCTGTC -3′.); *cdh5* (forward primer, 5′- GGCAATCAA CTGTGCTCTCC -3′; reverse primer, 5′- CTTCGTGGA GGAGCTGATCT -3′); *vWF* (forward primer, 5′- CCGGAAGCGACCCTCAGA- 3′; reverse primer 5′ - CGG TCAATTTTGCCAAAGATCT -3′) and *GAPDH* (forward primer, 5′- TGTGTCCGTCGTGGATCTGA -3′; reverse primer, 5′- TTGCTGTTGAAGTCG CAGGAG -3′). Real time PCR was performed on an ABI PRISM 7500 sequence detection system with SYBR GREEN PCR Master Mix (Applied Biosystems) according to the manufacturer's instructions. The PCR conditions were 94°C for 1 minute followed by 95°C for 30 s then 58°C for 40 s with a total of 35 cycles. All of the reactions were run in triplicate and were normalized to GAPDH. The relative differences in PCR results were calculated by using the comparative Ct-method (2-ΔΔCT).

### Rat critical-sized calvarial bone defect (CSD) model

The *in vivo* experimental protocol was reviewed and approved by the University at Buffalo Animal Care and Use Committee. Thirty male SD rats at eight-week old were used in this study. Rats underwent surgery under general anesthesia with the 5% isoflurane/O_2_ gas inspiration for induction and 1–2% isoflurane/O_2_ gas for maintenance by using a facial mask. Buprenorphine was given as an analgesic pre-surgery. The scalps covering the calvarial vault were shaved and scrubbed with betadine solution and infiltrated with 0.1–0.5 ml of a local anesthetic agent of 2% lidocaine (20 mg/ml) with 1:100000 epinephrine (0.01 mg/ml). An incision was made along the midline. Full-thickness skin and the periosteum were raised to expose the calvarial bone surface. An 8-mm-diameter trephine bur was used to drill a standardized, round, segmental defect around the sagittal suture. During drilling, the area was irrigated with saline solution and the underlying dura mater was maintained intact. A single transplant of 1×10^6^ cells mixed with nCS/A paste was injected into each defect. The periosteum (pericranium) and skin were closed in layers with non-absorbable 4–0 prolene sutures [47]. After surgery, the rats were treated with carprofen for 2 days to minimize pain or discomfort according to the protocol. Animals were divided into 5 groups randomly: group 1, nCS/A scaffold only; group 2, nCS/A+MSCs (nCS/M); group 3, nCS/A+MSCs+EPC (nCS/M+E); group 4, nCS/A+Ad-BMP2–MSCs (nCS/B2M), and; group 5, nCS/A+Ad-BMP2–MSCs+Ad-BMP2-EPCs (nCS/B2(M+E)). At the end of the 5 weeks following the surgery, animals were euthanized using CO_2_ and the calvaria bone was harvested for further analysis.

### Analysis of bone regeneration

Bone Density Measurements (BMD, g/cm^2^) were performed using LUNAR PIXImus bone densitometer and analyzed by LUNAR PIXImus software according to the equipment manual book. Total of six samples per groups were analyzed. On the computerized scan plots, five regions of interest (ROI) of each slide for six slides per group were selected to measure BMD of the defect area and the average of these values was taken as the final result. For histological analysis, half specimens (half implant) of six samples per group were decalcified and cut into 5 µm sections. The sections were then stained with hematoxylin and eosin (H&E) or for immunohistochemistry analysis. Digital images of each slide were acquired using a digital camera mounted to a microscope. Newly formed bone areas in the total scaffold area were calculated manually at 10 × magnification by using NIH ImageJ software. The other half specimens of six samples per group were used for ALP activity analysis using the ALP Assay Kit (Sigma) and DNA assay kit (Invitrogen) as previously described [43].

### Analysis of Blood Vessel Ingrowth

Paraffin-embedded decalcified bone sections were processed for immunohistochemistry staining for vWF and VEGF, which present in large quantities in sub-endothelial matrices, as described previously[42]. Three independent samples per groups were analyzed. Primary antibodies for goat wWF and goat VEGF were (Santa Cruz Biotechnology, Santa Cruz, CA) diluted at 1:300 ratio and secondary rabbit anti-goat antibody conjugated to HRP (Jackson ImmunoResearch Laboratories, Inc. West Grove, PA) was diluted at 1:500 ratio in 1% BSA. Peroxidase activity was visualized with diaminobenzidine. Negative controls and positive controls were included at the same time with same conditions (see Figure S1). The negative control staining was performed on the same bone slides without the primary antibody (replaced by goat serum). The positive control staining was performed on the kidney slides with the same primary and secondary antibodies. Images were acquired by using AxioImager software. Blood vessel numbers in the whole implanted area (blood vessel numbers/10^4^ pixel) for each sample (2 slides per each independent sample) indicated by vWF and VEGF staining, were counted manually at 10×magnification in the total defect area by using NIH Image J software.

### Statistical analysis

Statistical analysis was performed using SPSS-17.0 software. Where indicated, experimental data were reported as Mean ± SD of triplicate independent samples. Data were analyzed using Student's *t*-test and one-way analysis of variance (ANOVA), and Tukey's HSD test was applied as a post hoc test if statistical significance was determined. A value of *p≤*0.05 was considered statistically significant.

## Results

### Isolation and characterization of MSCs and EPCs

**Figure 1:**
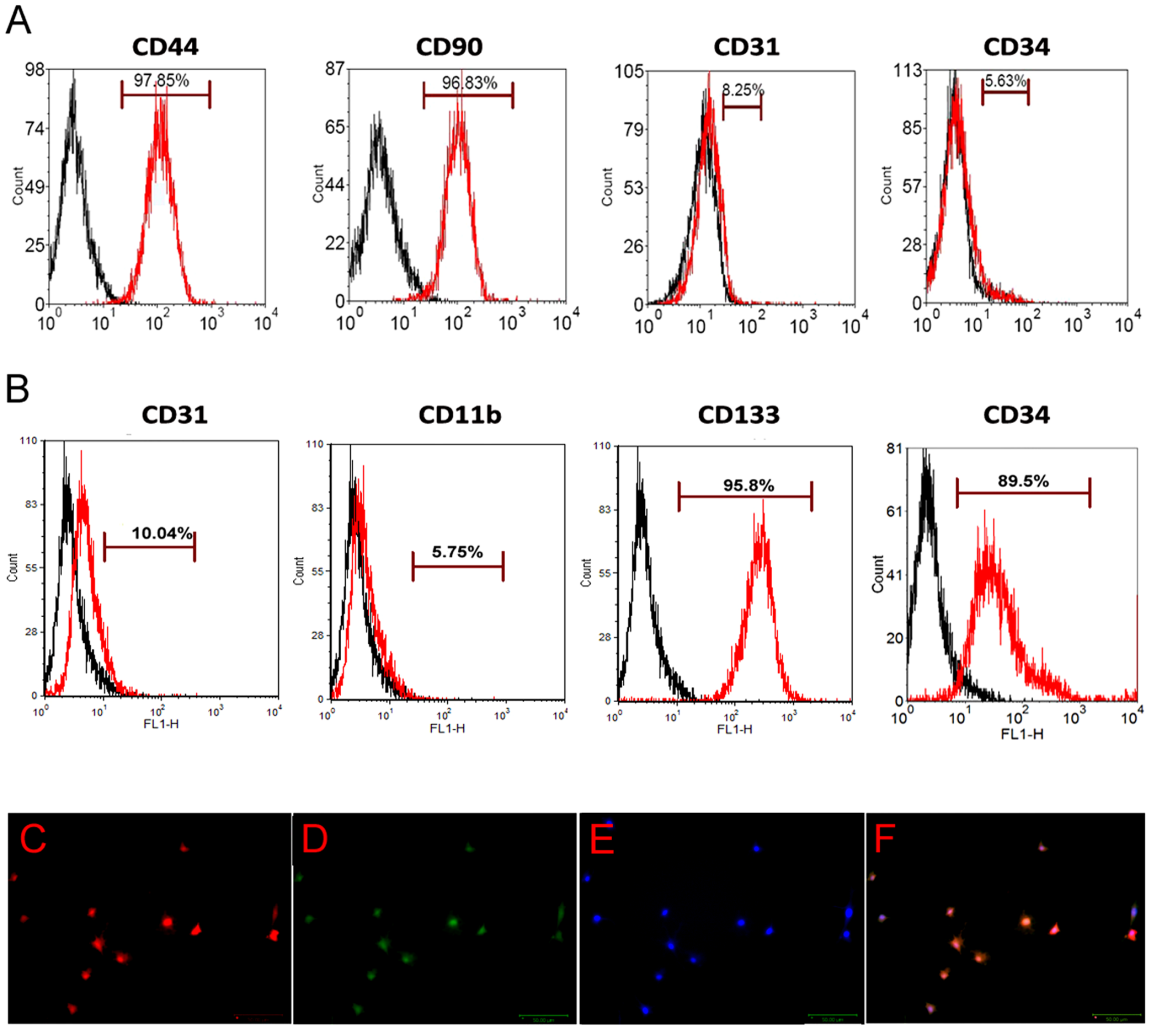
Phenotype identification of MSCs and EPCs. (A) Flow cytometry analysis for MSCs. The red open histograms indicated the cells which were stained positive for the undifferentiated MSCs markers -CD44 and CD90 and negative for CD 31 and CD34. The black open histograms showed isotype-matched control staining. (B) Flow cytometry analysis for EPCs. The red open histograms indicated the cells which were stained positive for the markers of undifferentiated EPCs - CD133 and CD34, and negative for CD31 and CD11b. The black open histograms showed isotope-matched control staining. (C-E) Fluorescence images of EPCs cultured for 14 days and stained with (C) Dil-Ac-LDL, (D) lectin and (E) DAPI. Bar = 50 µm. (F) Overlay of images of C, D and E.

The cells were analyzed and identified by flow cytometry analysis for MSC cell surface markers at passage 4. As shown in Figure 1A, MSCs expressed a cell-surface protein profile positive for CD44 (97.85%) and CD90 (96.83%) and negative for CD34 (5.63%) and CD31 (8.25%) [48].

For EPCs, we analyzed the early hematopoietic progenitor cell marker by flow cytometry. Results showed that EPCs expressed a cell-surface protein profile positive for CD133 (95.8%) and CD34 (89.5%), and negative for CD11b (5.75%) and CD31 (10.04%) (Figure 1B). To confirm EPC phenotype, we observed the expression of Dil-ac-LDL and the binding of Lectin by immunofluorescence staining. As shown in Figure 1 C-F, when the cells were cultured in EGM/OS media for 14 days, the cells were positive for Dil-ac-LDL (red) and lectin staining (green).

### Injectable nCS/A delivery system does not affect cell viability of MSCs and EPCs

**Figure 2:**
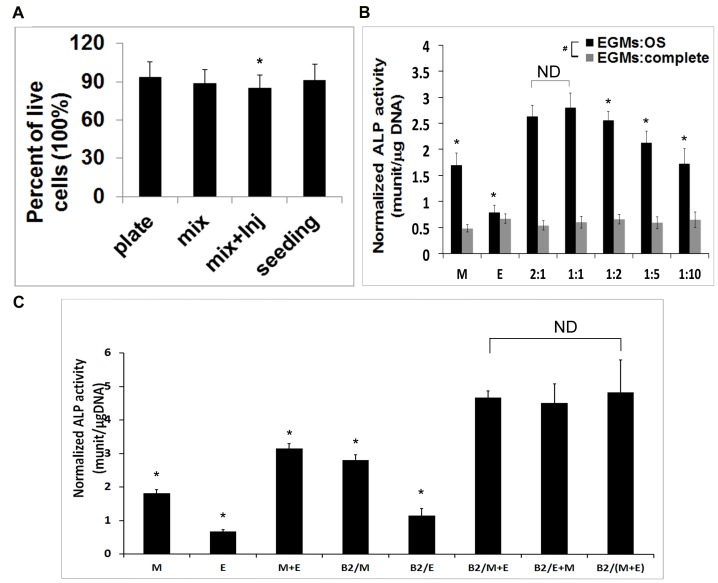
Co-culture of EPCs and MSCs in nCS/A scaffold. (A) MTS cell viability assay. The cell number was 5×10^4^ per scaffold for the four groups. Data represent the mean+SD for  = 8. *p*>0.05: between any two groups. (B) Optimization of the co-culture ratio of EPCs and MSCs. Cells were plated at EPCs:MSCs ratios of 0:1 (MSCs alone, indicate as M),1:0 (EPCs alone, indicate as E), 2:1 1:1, 1:2, 1:5 and 1:10 with a total cell number of 1×10^5^ and then cultured in EGM/CM (EGM media: complete media ratio of 1:1) or EGM/OS media (EGM media: OS media ration of 1:1) for 7 days for ALP activity assay. ND means no significant difference between each of those three groups (*p*>0.05). Significant difference from the group of 1:1 is indicated by *(*p*<0.05). # *p*<0.05: all other groups between the cells treated with EGM/CM and EGM/OS media except EPCs group, which has no difference between the cells treated with EGM/CM and EGM/OS media. (C) ALP activity. MSCs, EPCs, BMP2 gene-modified MSCs and BMP2 gene-modified EPCs were respectively plated in 24-well plates at a density of 2.5×10^4^ cells/cm^2^ in the different combination. The cells were induced with EGM/OS media (EGM media: OS media ration of 1:1) for 7 days for ALP assay. Data represent the mean+SD of *n* = 6 samples. In the groups, M: MSCs, E: EPCs, B2: BMP2, M+E: MSCs+EPCs, B2/M: BMP2 gene-modified MSCs, B2/M+E: EPCs and BMP2 gene -modified MSCs. B2/(M+E): BMP2 gene-modified MSCs and BMP2 gene-modified EPCs. ND means no significant difference between any two groups of those three groups (*p* >0.05). Significant difference from the group of B2/(M+E) is indicated by * (*p* <0.01).

To determine whether mixing MSCs and EPCs (1:1) with injectable nCS/A paste affects cell viability and proliferation, the MTS cell viability assay was performed. The results showed no significant differences in cell viability among those four groups, demonstrating that mixing and injecting MSCs and EPCs with the injectable nCS/A scaffold does not affect cell viability and proliferation (Figure 2A).

### Co-culture of BMP2 gene-modified EPCs and MSCs enhances osteoblast differentiation

To identify the best ratio for co-culture of MSCs and EPCs for osteogenic differentiation, we plated EPCs and MSCs at the following ratios (EPCs∶ MSCs = 0∶1, 1∶0, 2∶1, 1∶1, 1∶2, 1∶5 and 1∶10) and treated cells with EGM/complete media (CM) or EGM/OS media for 7 days prior to ALP activity assay. The ratios of 1∶1 and 1∶2 showed the much higher ALP activity (*p*<0.05) compared to any other ratios (Figure 2B). The ALP activity in 1∶1 group was slightly higher than that in 2∶1 group but there was no significant difference (*p*>0.05). Based on these results, we chose the 1∶1 ratio of MSCs and EPCs for the further studies. To test whether the combination of BMP2, MSCs and EPCs promotes osteoblast differentiation, we performed the ALP activity assay. As shown in Figure 2C, ALP activity in BMP2 gene-modified MSCs and EPCs group (B2/(M+E)) was about 4.3 fold, 9.6 fold, 2.3 fold and 1.9 fold higher than that in MSCs group (M), EPCs group (E), MSC with EPCs group (M+E) and BMP2 gene-modified MSCs group (B2/M). However, there was no significant difference among the B2/M+E, B2/E+M and B2/(M+E). These results demonstrated that the combination of BMP2, MSCs and EPCs enhances osteoblast differentiation.

### The combination of BMP2, MSCs and EPCs in nCS/A scaffold increases the expression of osteoblast and endothelial marker genes

**Figure 3:**
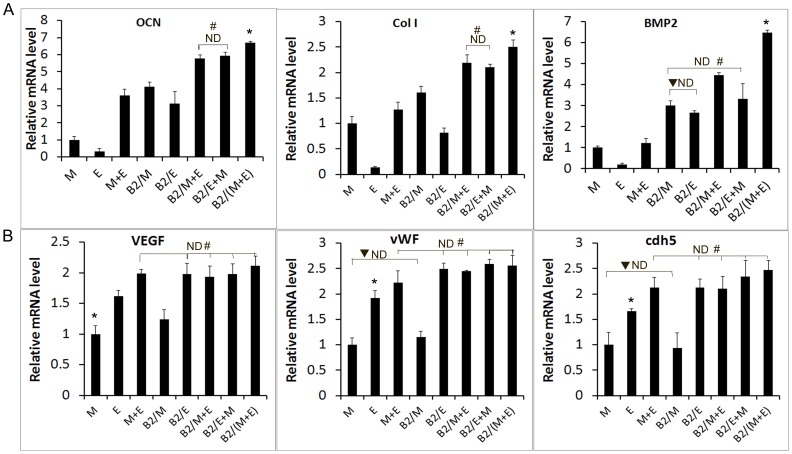
qRT-PCR analysis of osteoblast and endothelial marker genes. MSCs, EPCs or MSCs and EPCs with or without BMP2 gene modification (1∶1) were plated in 24-well plate as a density of 2.25×10^4^ cells/cm^2^ as described in *Materials and Methods*. These groups were MSCs alone (M), EPCs alone (E), MSCs +EPCs (M+E), BMP2 gene-modified MSCs (B2/M), BMP2 gene-modified EPCs (B2/E), EPC+BMP2 gene-modified MSCs (B2/M+E), MSC+BMP2 gene-modified EPCs (B2/E+M), and BMP2 gene-modified MSCs+BMP2 gene-modified EPCs (B2/(M+E)). The cells were induced with EGM/OS media for 14 days for qRT-PCR analysis. Data represent the mean+SD of *n* = 6 samples. (A) Osteoblast marker genes: OCN, Col I and BMP2. (B) Endothelial cell marker genes: VEGF, cdh5 and vWF. For A and B, ND: *p*>0.05, there is no significant difference between any two groups among the indicated ND groups. * *p*<0.05: between any two groups except the ND groups. # *p*<0.05: any ND groups vs. any other groups. ▾ *p*<0.05, any group in ND groups vs. any other groups.

To further investigate whether the combination of BMP2, MSCs and EPCs affects osteoblast and endothelial marker gene expression, *OCN*, *Col I* and *BMP2* (osteoblast marker genes) and *VEGF*, *cdh5* and *vWF* (endothelial marker genes) were analyzed by real time RT-PCR. The results showed that the expression levels of *OCN*, *Col I* and *BMP2* in B2/(M+E) were apparently higher than those in the other five groups- M, E, M+E, B2/M and B2/E. Compared with the M group, the combination of MSCs and EPCs can increase the expression of osteoblast marker genes. Moreover, with BMP2 gene modification, osteoblast marker gene expression was significantly up-regulated in all groups (Figure 3A). These results suggested that BMP2 could greatly promote osteoblast differentiation of MSC. In contrast, the mRNA levels of endothelial marker genes - *VEGF*, *cdh5* and *vWF* were markedly up-regulated in the groups containing EPCs (E, M+E, B2/E, B2/M+E, B2/E+M and B2/(M+E)) compared to the groups without EPCs (M and B2/M). Additionally, BMP2 transfection slightly up-regulated the levels of these genes in B2/E compared to the group without BMP2 (E). These results indicated that BMP2 also promotes the expression of endothelial marker genes and EPCs differentiation into endothelial cells, but to a lesser extend compared to its enhancement to osteogenesis.

### Injectable nCS/A delivery system with BMP2 gene-modified MSCs and EPCs promotes bone and blood vessel growth *in vivo*

**Figure 4:**
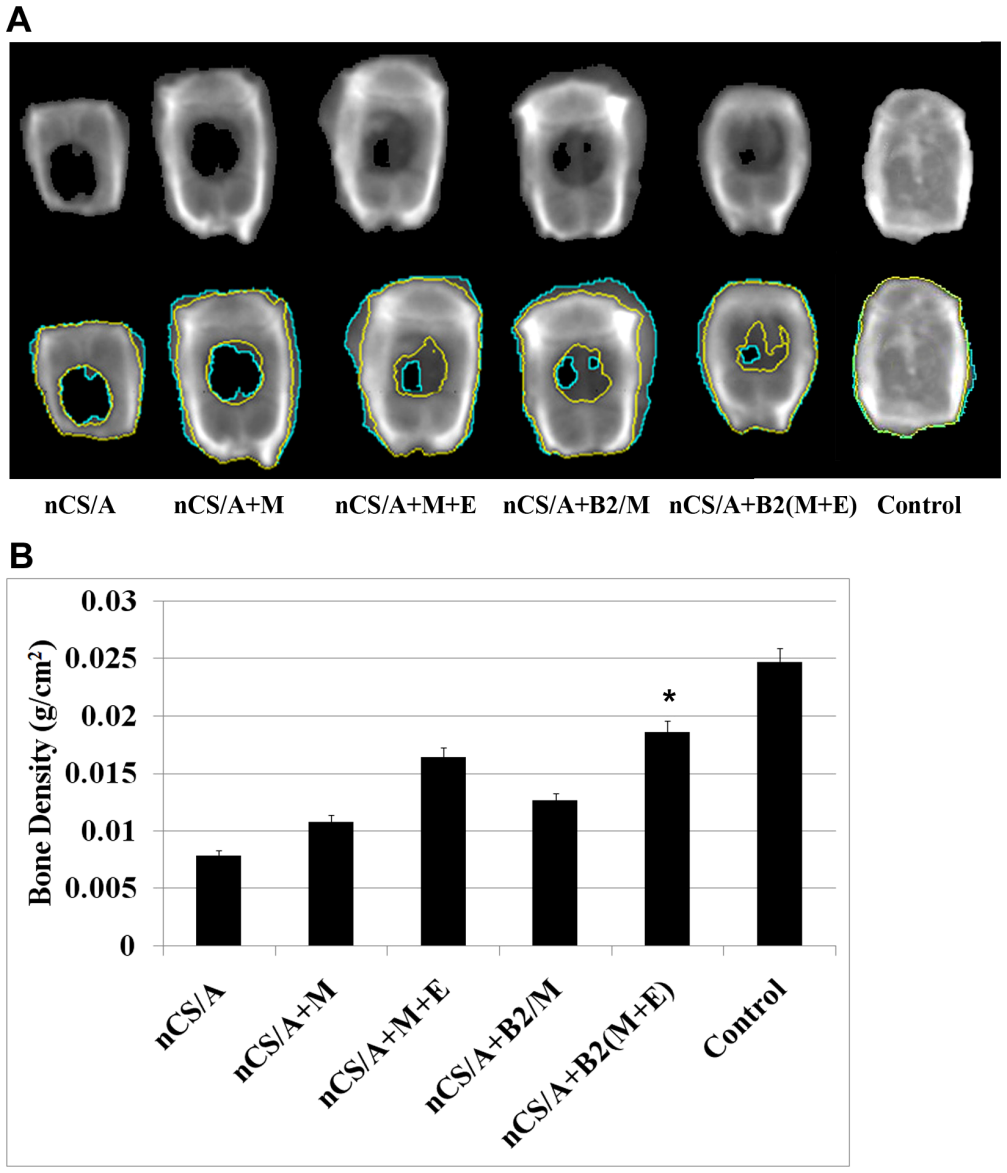
Bone mineral density analysis for bone growth in rat critical-sized calvarial defect model. (A) Images of calvarial bone obtained using LUNAR PIXImus system, 5 weeks after surgery. Upper row: general x-ray view. Lower row: x-ray with bone regeneration areas outlined in different colors. The black area circled with green lines in the implanted region is the low density area (fibrous tissue). The dark gray area between the green line and the yellow lines in the implanted region is the bone area with BMD that is lower than normal BMD. The area between the two yellow lines is considered the normal BMD area; bone density in this area is close to the BMD of normal bone tissue. (B) Quantitative analysis of bone density from A. The average BMD of normal intact bone is 0.0245 g/cm^2^. *p<0.05: nCS/A+B2/(M+E) vs. each of the other five groups.

To evaluate the potential of the injectable nCS/A delivery system and BMP2 gene-modified MSCs and EPCs for bone and vascular regeneration *in vivo*, 8-mm bone defects were created in the calvarial bones of 8-week-old SD rats. This critical-sized defect cannot spontaneously heal during bone healing period [47]. The cranial bones were harvested at 5 weeks. BMD tests showed that the BMP2 gene-modified MSCs+EPCs group (nCS/A+B2/(M+E)) exhibited robust osteogenic activity, with nearly complete closure of bony defects. The MSCs+EPCs (nCS/A+M+E) group showed less normal bone density area than nCS/A+B2/(M+E) group. However, it showed more normal bone density area than the BMP2 gene-modified MSCs group (nCS/A+B2/M) as shown in Figure 4A, indicating that the combination of EPCs and MSCs promotes bone regeneration. Quantitative analysis of bone mineral density (BMD) showed that the BMD in nCS/A+B2/(M+E) was significantly higher than that in the other four groups (Figure 4B). Without BMP2 gene transduction, the BMD in the nCS/A+M+E group was also significantly higher than that in nCS/A+M group and close to that in the nCS/A+M/B2 group (Figure 4A, B). 74% of BMD in the control group with normal intact calvarial bone, indicating

**Figure 5:**
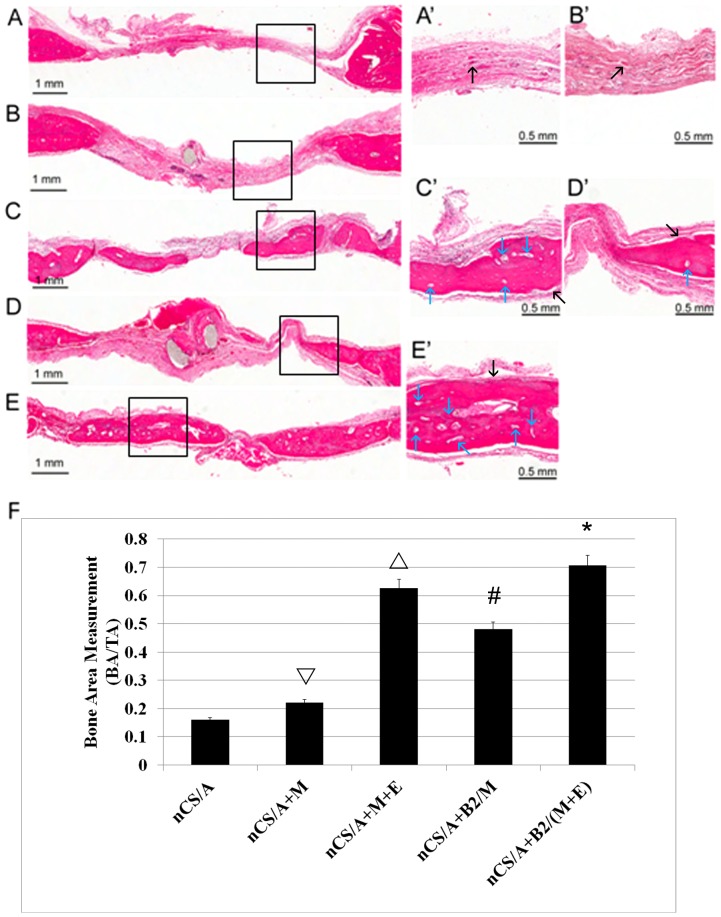
Bone growth in rat critical-sized calvarial defect model. (A-E and A’-E’) Hematoxylin and eosin staining of calvarial bone for analysis of the new bone formation. Coronal sections through the midline of defects are shown. Margins of the original 8.0 mm trephine defect are shown. (A-E) lower magnification, bar = 1 mm. (A’-E’) higher magnification, bar = 0.5 mm. (A, A’): nCS/A; (B, B’): nCS/A+M; (C, C’): nCS/A +M+E; (D, D’): nCS/A+B2/M; (E, E’): nCS/A+B2/(M+E). Black arrow: newly formed bone. Blue arrow: blood vessels. (F) Quantitative analysis of bone area in implanted region from (A-E). BV, bone area in the implant; TV, total implant area. *p<0.05: nCS/A+B2/(M+E) vs. each of the other four groups. #*p*<0.05; nCS/A+M+E vs. nCS/A+B2/M or nCS/A+M or nCS; ▵*p*<0.05: nCS/A+B2/M vs. nCS/A+M or nCS/A only; ▿*p*<0.05: nCS/A+M vs. nCS/A.

Hematoxylin and eosin-stained sections showed that at 5 weeks following the implantation, no residual materials or inflammatory infiltrating cells were seen within any of the defect regions. In all samples, the smallest amount of new bone (black arrows) was formed in the nCS group compared with other groups, and most of defect areas were covered by fibrous like tissues (Figure 5A and A’). The nCS/A+M group formed small amounts of new bone, and most of defect areas were also covered by fibrous like tissues (Figure 5B and B’). The nCS/A+B2/M and nCS/A+M+E groups had much more bone formed in the defect area with partial coverage of the defect areas compared with nCS and nCS/A+M group (Figure 5C, C’ and 5D, D’), while nCS/A+B2/(M+E) group exhibited robust osteogenic activity, and new bone was continuous and covered almost all defect areas (Figure 5E and E’). Histomorphometric analysis showed that the amount of newly-formed bone (BA) to the total implant area (TA) in the nCS/A+B2/(M+E) group was significantly greater than that in the other four groups (*p<0.05*) (Figure 5F). Without BMP2 gene transduction, BA/TA in nCS/M/E were also significantly higher than in the nCS/M/B2, nCS/M or nCS groups, indicating EPCs could promote bone regeneration. There was significantly higher BA/TA among those groups treated with nCS/M/B2 and nCS/M, as compared to those treated with nCS/M and nCS. Notably, we also found there were much more blood vessels in the groups with EPCs compared to those without EPCs (Figure 5, blue arrows).

**Figure 6:**
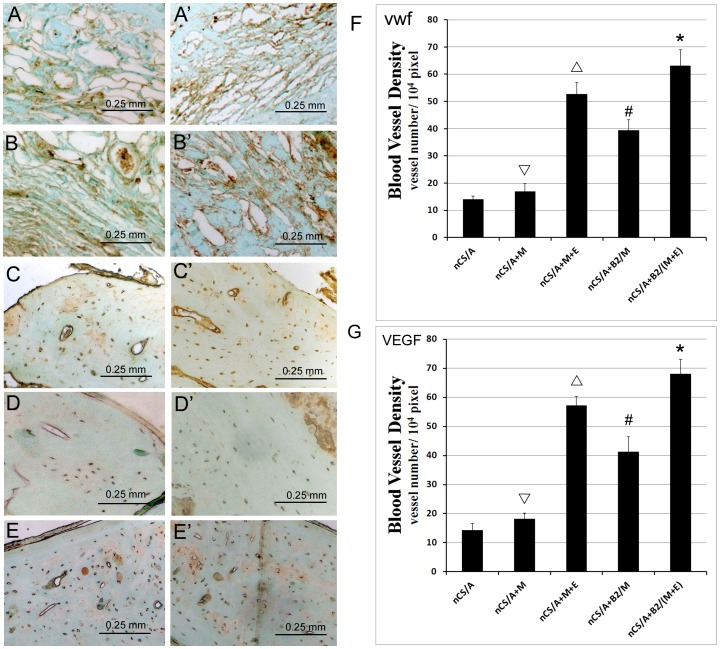
Blood vessel growth in rat critical-sized calvarial defect model. (A-E) Immunostaining of endothelial cell marker - vWF. (A’-E’) Immunostaining of VEGF. (A, A’): nCS/A; (B, B’): nCS/A+M; (C, C’): nCS/A+M+E; (D, D’): nCS/A+B2/M; (E, E’): nCS/A+B2/(M+E). (F) Quantitative analysis of blood vessel density from (A-E). (G) Quantitative analysis of blood vessel density from (A’-E’). *N* = 5, bar = 0.25 mm. *p<0.05: nCS/A+B2/(M+E) vs. each of the other four groups. #*p*<0.05: nCS/A+M+E vs. nCS/A+B2/M or nCS/A+M or nCS; ▵*p*<0.05: nCS/A+B2/M vs. nCS/A+M or nCS/A only; ▿*p*<0.05: nCS/A+M vs. nCS/A.

To confirm the vascular formation in the implants, immunostaining with anti-vWF (Figure 6A-E) and VEGF (Figure 6A’-E’) antibodies was performed, which could specifically stain vascular endothelial cells (Fig S1 and Fig S2). As shown in Figure 6A, much more blood vessels (brown color) were found in the groups of nCS/A+B2/(M+E) and nCS/A+M+E compared to the groups of nCS/A+B2/M, nCS/A+M and nCS/A. Quantification of blood vessel densities throughout the total implant area (BVD) confirmed that nCS/A+B2/(M+E) group had a significantly higher BVD than other groups (Figure 6F). These results demonstrate that EPCs can promote vascular growth, and that the combination of BMP2, MSCs and EPCs dramatically increases vascularized bone regeneration.

## Discussion

It is believed that efficient regeneration of bone defects can be achieved by the combination of three regenerative elements - a scaffold, cells, and growth factors. As scaffolds, a number of biomaterials, such as bioceramics, biopolymers, and synthetic polymers, have been innovated, improved, and applied clinically [49]. MSCs and EPCs have been recently established as potential components for use in tissue regeneration and repair [50]. In order to design more advanced scaffold systems which will enable bone defect healing with less pain, fewer scars, decreased morbidity and less disruption of the soft tissue envelope, the scaffolds should be designed to be biodegradable, moldable and injectable. In our previous study, we have developed a novel injectable nCS/A stem cell delivery system. We further found that there is no apparent lymphocyte infiltration in the implanted regions indicating that this system has no apparent immune response in rat calvarial bone defect model [38]. The innovation of this study is to develop and investigate a multi-stem cell scaffold system for healing CSBDs by combining the injectable and biodegradable nCS/A scaffold and BMP2 genetically engineered MSCs and EPCs. Our results for the first time demonstrated that this multi-stem cell system promotes osteoblast and endothelial cell differentiation *in vitro* and significantly accelerates bone healing in CSBDs *in vivo* by supporting vascularized bone regeneration.

EPCs and MSCs have arisen as potentially useful cells for neovascularization and tissue repair and regeneration. EPCs originate in the hematopoietic compartment of the bone marrow and are a heterogeneous group of endothelial cell precursors. EPCs can also be isolated from peripheral blood as well as the spleen. MSCs are fibroblast-like cells which can be isolated from a variety of tissues, such as bone marrow, periosteum, trabecular bone, adipose tissue, synovium, skeletal muscle, and dental pulp. To date, specific markers for each cell type remain lacking. A number of surface proteins have been used to enrich rat MSCs, including CD44, CD90, CD73, CD105, CD271 and Stro-I [51], [52]. Here, we used CD44 and CD90 as positive markers to enrich MSCs. The HSC marker CD31 and EPCs marker CD34 were used to confirm that the MSCs were depleted of HSCs and EPCs [53]. Our results showed that MSCs expressed a cell-surface protein profile positive for CD44 and CD90 and negative for CD31 and CD34 [48]. For the EPCs, we detected the early hematopoietic progenitor cell marker, CD133 (AC133), which is not expressed after differentiation. [54], [55]; CD34, which is expressed in EPCs [20], [56], and CD 31, which is negligibly expressed in EPCs but highly expressed in mature endothelial cells [57], as well as CD11b, which is expressed in monocytes, but not in EPCs [58]. Our results showed that EPCs expressed a cell-surface protein profile positive for CD133 and CD34 and negative for CD11b and CD31. These results confirmed the rat MSC and EPC phenotypes and suggested that the above markers should be suitable for identification of rat MSCs and EPCs.

MSCs genetically modified to produce bone morphogenetic protein 2 (BMP2) have been previously proved to enhance bone regeneration in mice [59]. Our previous study demonstrated that the combination of nCS/A injectable scaffold and BMP2 gene-modified MSCs can promote bone regeneration [42]. However, these strategies cannot successfully heal CSBDs due to inefficient blood supply [60], which results in hypoxia and insufficient supply of nutrients and removal waste products of metabolism [61]. To overcome this problem, in this study we combined an injectable nCS/A scaffold, BMP2, MSCs and EPCs for promoting vascularized bone regeneration. Our results from *in vivo* densitometric scans showed much higher BMD in defect areas in the groups with EPCs compared to the groups without EPCs. Histologic analysis complemented the BMD results, showing that more bony tissue and blood vessel formation in the groups with EPCs. Moreover, immunohistochemistry analysis verified those findings, showing that with EPCs, the blood vessel density in the defect areas was higher than that in the groups without EPCs. In contrast, the lack of EPCs resulted in a relatively reduced vascularization and bone formation. One possible mechanism is that simply relying on ingrowth of the host vessels is inadequate for bone regeneration due to the large distance between the host tissue blood vessels and the center of bone defect. As a result, nutrients, metabolites, and other molecules cannot be transported into the defect central region, preventing bone regeneration. Whereas in the groups with EPCs, the implanted EPCs directly increase vascular ingrowth and/or recruit host EPCs and MSCs migration into the defect areas, which leads to the greater availability of nutrients, cytokines and other molecular factors involved in the bone healing process. This mechanism is supported by the previous studies. For example, Asahara et al reported that the application of EPCs could initiate and facilitate neovascularization [19]. Kaigler et al. reported that the transplanted endothelial cells enhance orthotopic bone regeneration in a calvarial defect [62]. Also further studies showed that implantation of EPCs and MSCs improves heterotopic ossification [63] and orthotopic bone regeneration [24]. Most recently, Seebach et al found that EPCs and EPCs/MSCs Loaded β-TCP improve early vascularization by directly forming new vessels and stimulating host EPCs and MSCs migration into the implanted regions, which led to an improved vascularization in bone regeneration[64]. Thus, these findings highlighted that vascularization in implants play a critical role in bone defect healing especially for CSBDs.

Most notably, we found that with BMP2 gene transfection, a combination of MSCs and EPCs could increase the expression of osteoblast and endothelial marker genes *in vitro* and significantly promote the blood vessel and ectopic bone formation in a CSBD model compared to the groups without BMP2. Most area of CSBDs (about 70%) in the B2(M+E) group were filled with the regenerated bone in a short healing time – 5 weeks. This result can be reasonably explained, because BMP receptors are expressed in EPCs and MSCs, and BMP2 not only stimulates MSCs to differentiate into osteoblasts and increase VEGF expression as supported by our co-culture studies, but also induces EPCs in a dose-dependent activation of chemotaxis [28]. Meanwhile, increased VEGF stimulates EPC differentiation into endothelial cells and vascularization [65]. Additionally, a recent study reported that endothelial cells could produce BMP-2 during bone and vascular formation in a model of distraction osteogenesis. This finding suggests that endothelial cells may play an important role in stimulating osteogenesis for bone repair [66]. Thus, exogenous up-regulation of BMP-2 production in EPCs may also explain the significant increase in bone formation in the groups with EPCs. Collectively, these results suggest that the construction with BMP2 gene -modified MSCs and EPCs likely facilitates the coupling between vasculogenesis and osteogenesis during bone regeneration.

## Conclusions

In summary, this study provides evidence that the combination of nCS/A scaffolds with BMP2 gene-modified MSCs and EPCs promotes osteoblast and endothelial cell differentiation *in vitro* and significantly accelerates bone healing in CSBDs *in vivo* by supporting vascularized bone regeneration. On the basis of the data presented here, we conclude that this system has the potential to provide reconstructive surgeons with new and advanced treatment modalities for wounded patients with CSBDs by promoting vascularized bone regeneration with fewer surgeries, reduced pain and shorter healing time compared to current best practices.

## Supporting Information

Figure S1 **Negative and positive controls for the immunostaining.** The negative control staining was performed on the same bone slides under the same conditions except using the goat serum to replace the primary antibodies of anti- vWF (A) and anti-VEGF (A’). The positive control staining was performed on the kidney slides with the same primary and secondary antibodies under the same conditions (B, anti-vWF; B’, anti-VEGF).
(TIF)

Figure S2 **Higher magnification image of immunostaining.** (A-E) Immunostaining of endothelial cell marker - vWF. (A’-E’) Immunostaining of VEGF. (A, A’): nCS/A; (B, B’): nCS/A+M; (C, C’): nCS/A+M+E; (D, D’): nCS/A+B2/M; (E, E’): nCS/A+B2/(M+E). Bars: 0.25 mm.
(TIF)
