# Supplementary figures and images for: BMP2 Genetically Engineered MSCs and EPCs Promote Vascularized Bone Regeneration in Rat Critical-Sized Calvarial Bone Defects
Source: PLoS One. 2013 Apr 2;8(4):e60473. doi: 10.1371/journal.pone.0060473 (PMC3614944; doi:10.1371/journal.pone.0060473)

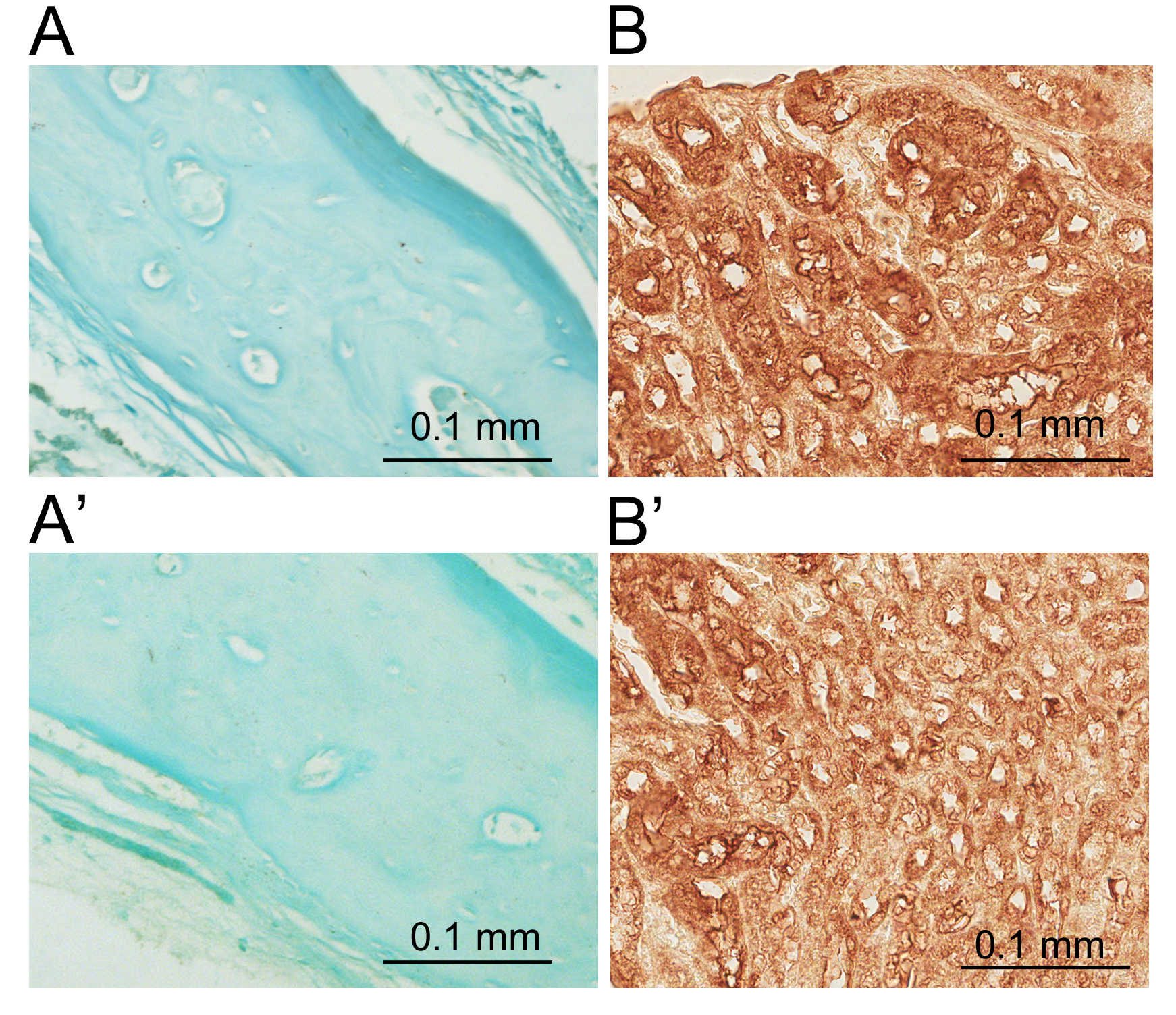

Supplement: Figure S1 — Negative and positive controls for the immunostaining. The negative control staining was performed on the same bone slides under the same conditions except using the goat serum to replace the primary antibodies of anti- vWF (A) and anti-VEGF (A’). The positive control staining was performed on the kidney slides with the same primary and secondary antibodies under the same conditions (B, anti-vWF; B’, anti-VEGF). (TIF) [file pone.0060473.s001.tif]

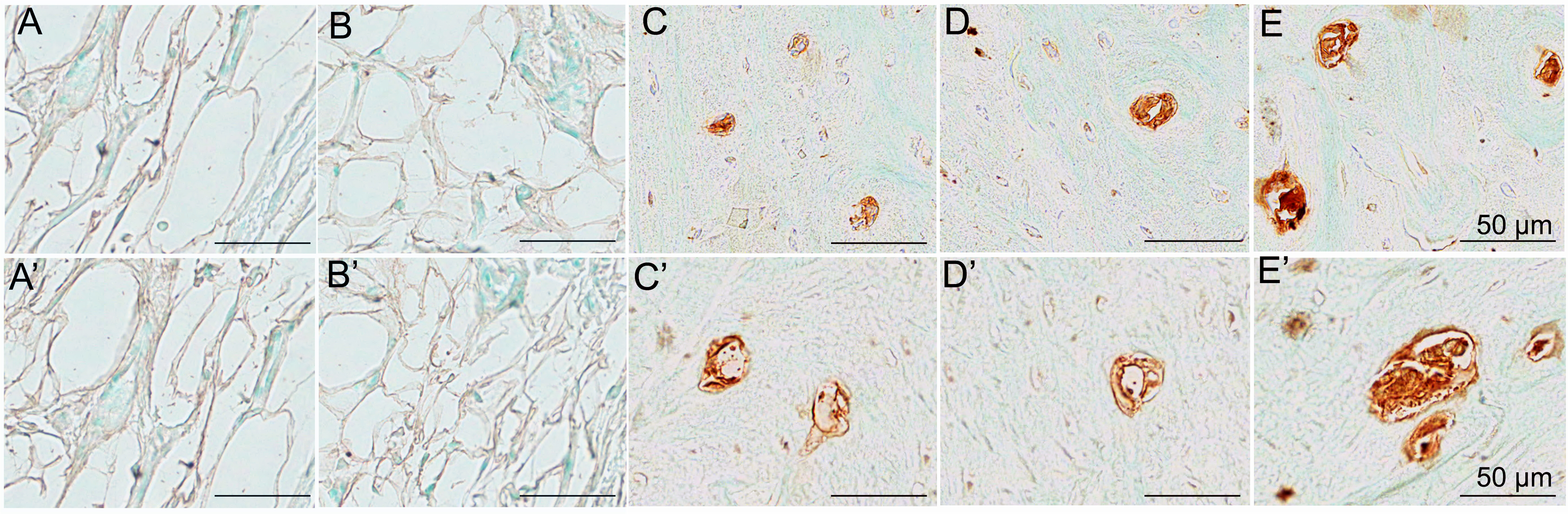

Supplement: Figure S2 — Higher magnification image of immunostaining. (A-E) Immunostaining of endothelial cell marker - vWF. (A’-E’) Immunostaining of VEGF. (A, A’): nCS/A; (B, B’): nCS/A+M; (C, C’): nCS/A+M+E; (D, D’): nCS/A+B2/M; (E, E’): nCS/A+B2/(M+E). Bars: 0.25 mm. (TIF) [file pone.0060473.s002.tif]
